# Supplementary material for: Trichostatin D as a Novel KLF2 Activator Attenuates TNFα-Induced Endothelial Inflammation
Source: Int J Mol Sci. 2022 Nov 3;23(21):13477. doi: 10.3390/ijms232113477 (PMC9656815; doi:10.3390/ijms232113477)
Supplement: Supplementary file 1 [file ijms-23-13477-s001.zip › ijms-1962379-supplementary.pdf]

Supplementary Data

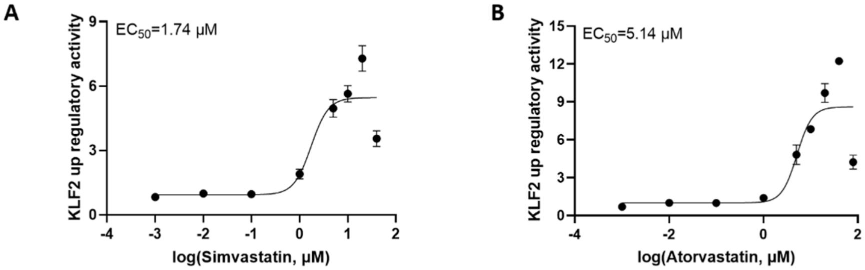

Supplementary Figure S1. Simvastatin and Atorvastatin were KLF2 activators. **(A, B)** KLF2 dose-response curve of Simvastatin **(A)** and **(B)**. COS-7 cells were transfected with a KLF2-Luc plasmid for 6 h and then treated with the vehicle (0.1% DMSO) or Simvastatin (0.001, 0.01, 0.1, 1.0, 5, 10, 20, 40, and 80 μM) for 24 h. Then, the activity of the KLF2 luciferase reporter gene was analyzed. Values are represented as the mean ± SEM; n = 3.

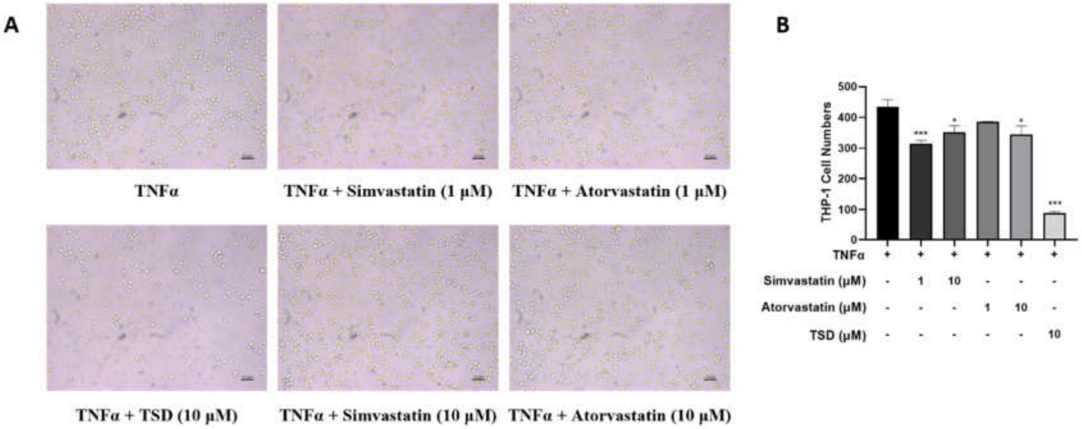

Supplementary Figure S2. Simvastatin and Atorvastatin attenuated monocyte adhesion to HUVECs. **(A, B)** Representative images (**A**) and quantification results **(B)** of monocyte adhesion to HUVECs after treatment with Simvastatin, Atorvastatin, and TSD. The cells were plated in 6-well plates and treated with Simvastatin (1 μM and 10 μM), Atorvastatin (1 μM and 10 μM), and TSD (10 μM) or DMSO for 18 h; then, TNFα (10 ng/mL) was added to the cells for 6 h. THP-1 cells were co-incubated for another 30 min. Values are represented as the mean ± SEM; n = 3; one-way ANOVA was used for analysis. *$p < 0.05$, *** $p < 0.0001$.
